# *Lamium album* Flower Extracts: A Novel Approach for Controlling *Fusarium* Growth and Mycotoxin Biosynthesis

**DOI:** 10.3390/toxins15110651

**Published:** 2023-11-13

**Authors:** Pascaline Aimee Uwineza, Monika Urbaniak, Łukasz Stępień, Anna Gramza-Michałowska, Agnieszka Waśkiewicz

**Affiliations:** 1 Department of Chemistry, Poznań University of Life Sciences, Wojska Polskiego 75, 60-625 Poznań, Poland; agat@up.poznan.pl; 2 Pathogen Genetics and Plant Resistance Department, Institute of Plant Genetics, Polish Academy of Sciences, 60-479 Poznań, Poland; murb@igr.poznan.pl (M.U.); lste@igr.poznan.pl (Ł.S.); 3 Department of Gastronomy Science and Functional Foods, Poznań University of Life Sciences, Wojska Polskiego 31, 60-624 Poznań, Poland; anna.gramza@up.poznan.pl

**Keywords:** white dead nettle, supercritical fluid extraction, integrated pest management, plant extracts, phytopathogenic, anti-mycotoxigenic, ergosterol

## Abstract

*Lamium album* is a medicinal flowering plant that is rich in bioactive compounds with various biological properties. *Fusarium* species, known for causing significant crop losses and mycotoxin contamination, pose threats to food safety and human health. While synthetic fungicides are commonly employed for fungal management, their environmental impact prompts the ongoing development of alternative methods. This study aimed to evaluate the efficacy of *L. album* flower extracts in inhibiting the in vitro growth and biosynthesis of mycotoxins by *Fusarium culmorum* and *F. proliferatum* strains. The extracts were obtained by supercritical fluid extraction using CO_2_ (SC-CO_2_). The effects of various concentrations (2.5, 5, 7.5, and 10%) were assessed on a potato dextrose agar (PDA) medium using the “poisoning” technique. *L. album* flower extracts reduced mycelium growth by 0 to 30.59% for *F. culmorum* and 27.71 to 42.97% for *F. proliferatum*. Ergosterol content was reduced by up to 88.87% for *F. culmorum* and 93.17% for *F. proliferatum*. Similarly, the amounts of synthesized mycotoxins produced by both strains were also lower compared to control cultures. These findings are a preliminary phase for further in vivo tests planned to determine the fungistatic effect of *L. album* flower extracts on cereal substrates as seedlings incubated in controlled environments and under field conditions. Their phytotoxicity and biological stability, as well as the possibility of formulating a bio-preparation to protect cereals against *Fusarium* infections, will be evaluated.

## 1. Introduction

Plant diseases represent a significant constraint for global crop yield, and fungal infections account for 80% of these losses [1]. *Fusarium* species are significant phytopathogenic and mycotoxigenic fungi that cause devastating effects on the global market, food safety, and farmers’ livelihoods [2,3]. Members of this genus infect a variety of staple cereals, including wheat, oat, barley, and maize, causing wilting, yellowing, browning, necrosis, and deformation. These fungi use several mechanisms to infect and colonize plants, including the production of enzymes that allow them to penetrate the plant cell wall. The production of mycotoxins that damage plant cells and enhance the pathogen’s ability to infect and colonize the plant is equally important [1,2]. Therefore, understanding these strategies is crucial for controlling *Fusarium* pathogens in plants.

*Fusarium culmorum* and *F. proliferatum* are polyphagous fungi that infect various plant species, particularly cereals, leading to diseases such as *Fusarium* head blight and *Fusarium* ear rot [4,5,6,7]. These diseases cause yield losses, reduce grain quality, and cause mycotoxin contamination, posing risks to human and animal health [7]. Mycotoxin contamination can occur at all stages of plant growth, influenced by local climate and the co-existence of multiple fungi [8,9,10]. Mycotoxins produced by these fungi, including deoxynivalenol (DON), nivalenol (NIV), zearalenone (ZEN), and fumonisins from B group (FB_1_, FB_2_, and FB_3_), have detrimental effects on organisms [3,8,11,12]. They also pose challenges to food security by reducing crop yield, increasing food prices, and impacting global markets and low-income consumers [7]. Controlling these mycotoxins is essential to prevent economic losses, ensure food safety, and address import restrictions [13,14]. Therefore, research continues to explore sustainable techniques for protecting and controlling fungal pathogens (primary source of mycotoxins in food and feed).

In response to those challenges, various initiatives have been developed [2,7,8]. These efforts include establishing legal limits for mycotoxins in food and feed, promoting sustainable pesticide use, and investing in research and development to identify alternative control strategies. Among them, using plant-based products, such as natural plant extracts and essential oils, is a promising alternative for synthetic fungicides in sustainable agriculture [13]. These solutions offer multiple benefits, including environmental and human health safety, minimal risk of resistance, and compatibility with organic and biodegradable farming practices [15,16]. Plant extracts contain bioactive compounds that disrupt various metabolic processes in fungi, such as the biosynthesis of ergosterol, a crucial sterol present in fungal cell membranes that plays a vital role in fungal growth [12,15,16,17,18,19].

*Lamium album*, commonly known as white dead nettle or non-stinging nettle, is a medicinal flowering herbaceous plant native to Europe, Western Asia, and North Africa [20]. This plant’s young shoots, leaves, and flowers are edible (fresh or cooked). Nowadays, *L. album* is used to make beverages such as tea and dietary supplements that claim to detoxify the organism and prevent menstrual disorders, abdominal inflammation, and musculoskeletal diseases [21]. It is known for its anti-inflammatory, astringent, anti-septic, antibiotic, and bacteriostatic properties [22]. Various in vitro and in vivo model systems were used to reveal its antiviral, antibacterial, antioxidant, anticancer, cytoprotective, wound-healing, and other significant pharmacological effects [22,23,24,25,26,27].

Czerwińska et al. (2020) have identified iridoids (lamalbid), phenolic acids/depsides (chlorogenic acid), phenylpropanoids (verbascoside), and flavonoids (rutin, quercetin malonylhexoside, tiliroside) in aqueous and ethanolic-aqueous extracts of *L. album* [24], rich in phenolic acids and flavonoids, which are the main bioactive compounds responsible for multiple biological activities. Pourmirzaee et al. (2019) have also reported other significant pharmacological effects of *L. album* extracts, further supporting their potential as a valuable source of bioactive compounds with diverse health-related benefits [28,29]. However, as far as we know, there has been limited exploration of *L. album* flower extracts as a source of natural antifungal agents in agriculture. Additionally, no studies have been conducted on the efficacy of *L. album* flower extracts against *Fusarium* pathogens.

Therefore, this study was designed to assess the in vitro effects of *L. album* flower extracts against *F. culmorum* and *F*. *proliferatum*, as well as their ability to suppress mycotoxin production. One of the most innovative aspects of this study is the evaluation of the antifungal effects of the aerial part of *L. album* against agriculturally important cereal pathogens.

## 2. Results

### 2.1. Inhibitory Effect of L. album Flower Extract against Fusarium culmorum and F. proliferatum

**Figure 1:**
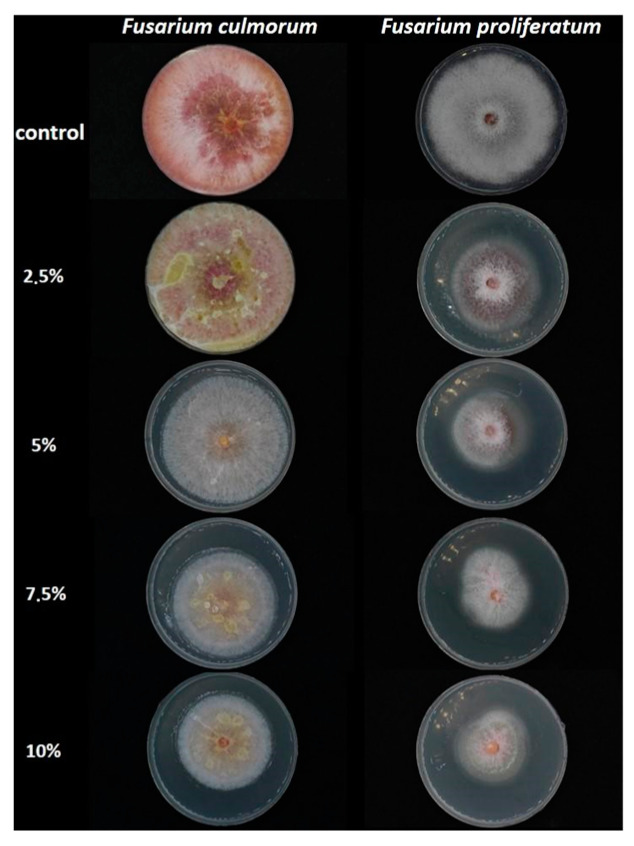
Petri dishes image showing the inhibitory effects of *L. album* flower extracts (2.5–10%) on mycelia growth of *F. culmorum* and *F. proliferatum* in the PDA medium after a 10-day incubation period.

**Figure 2:**
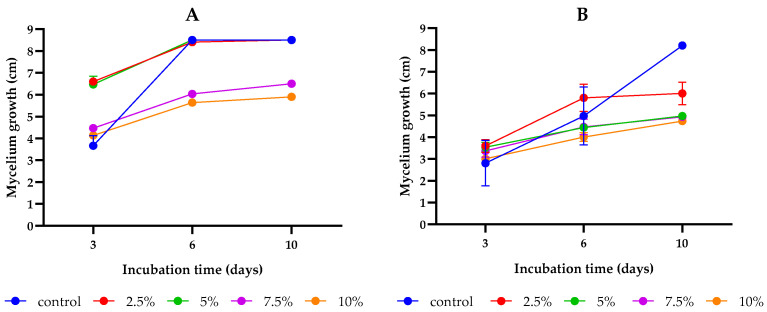
The impact of different concentrations (2.5, 5, 7.5, and 10%) of *L. album* on the mycelium growth of the studied *Fusarium* strains cultivated on PDA media over a 10-day incubation period: (**A**) *F. culmorum*, (**B**) *F. proliferatum*. The results are averages of three repetitions ± standard deviation.

This study aimed to assess the antifungal activity of SC-CO_2_ *L. album* flower extracts (2.5, 5, 7.5, and 10%) as compared to the control group (PDA without extract). After 10 days of incubation, an inhibitory effect of the studied concentrations of *L. album* flower extract was observed compared to the controls of both strains (Figure 1 and Figure 2). During the initial days of incubation, the fungistatic effect of the extract (2.5% and 5%) on *F. culmorum* diminished as early as day 3. However, by the final day of incubation, an inhibitory effect on colony growth became evident for 7.5% and 10% concentrations. Conversely, the antifungal effects of all studied concentrations on *F. proliferatum* were initially similar during the early stages of incubation. However, by the sixth day of incubation, the inhibitory effects of *L. album* began to manifest, and by the last day of incubation (tenth), a positive effect of all concentrations was observed as compared to the control. In addition, the inhibitory effects varied across different extract concentrations for each strain (Table 1). For *F. culmorum*, there was no discernible growth inhibition at lower extract concentrations of 2.5 and 5% (0% inhibition). As the extract concentration increased to 7.5% and 10%, inhibitory activity gradually emerged, resulting in inhibition percentages of 23.53% and 30.59%, respectively. However, for the *F. proliferatum* strain, all tested extract concentrations reduced the mycelium growth compared to the control. Significant differences were observed between the 2.5% concentration and the remaining concentrations (5, 7.5, and 10%); however, the increase in the extract concentrations from 5 to 10% did not differ significantly. Similarly to *F. culmorum*, the highest growth inhibition of 42.97% was observed for *F. proliferatum* at the highest tested concentration (10%).

### 2.2. The Antifungal Effects of L. album Flower Extract on the Ergosterol Content

The antifungal efficacy of *L. album* flower extract on *F. culmorum* and *F. proliferatum* growth was evaluated by measuring the ERG concentration using ultra-performance liquid chromatography with a photodiode array detector (UPLC/PDA) technique. In both strains examined, the application of *L. album* flower extracts considerably decreased the ERG content compared to the control (Table 2).

The results of the UPLC/PDA analysis (Table 2) showed that when the concentration of *L. album* flower extract increased, there was a significant reduction in ergosterol production by the studied strains, i.e., there was a dose-dependent response of *F. culmorum* and *F. proliferatum* to the *L. album* extracts. At the lowest concentration tested (2.5%), the extract reduced the ergosterol content by an average of 24.17% for *F. culmorum* and 61.56% for *F. proliferatum*. As the concentration of the extracts increased to 5, 7.5, and 10%, the decrease in ergosterol became more pronounced, with the highest concentration (10%) showing the most significant inhibition of ergosterol biosynthesis, with an average of 88.87% for *F. culmorum* and 93.17% for *F. proliferatum* in relation to the control.

### 2.3. The Effects of L. album Flower Extract on Mycotoxin Biosynthesis

**Figure 3:**
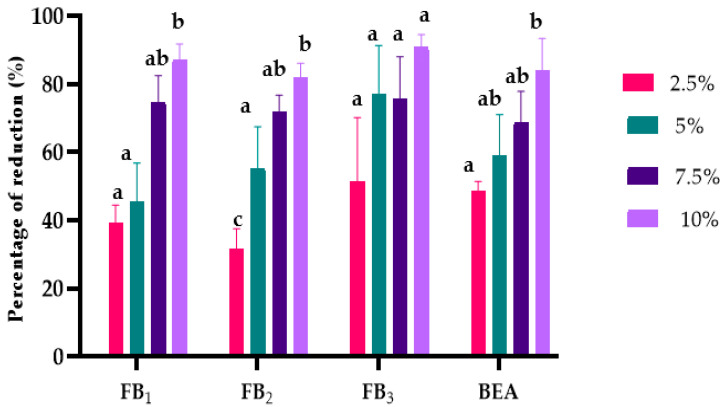
Effects of *L. album* extracts (2.5, 5, 7.5, and 10%) on reducing mycotoxins produced by *F. proliferatum*. The average with different letters (a–c) for each mycotoxin is significantly different at *p* < 0.01. Error bars represent standard deviation (n = 3).

**Figure 4:**
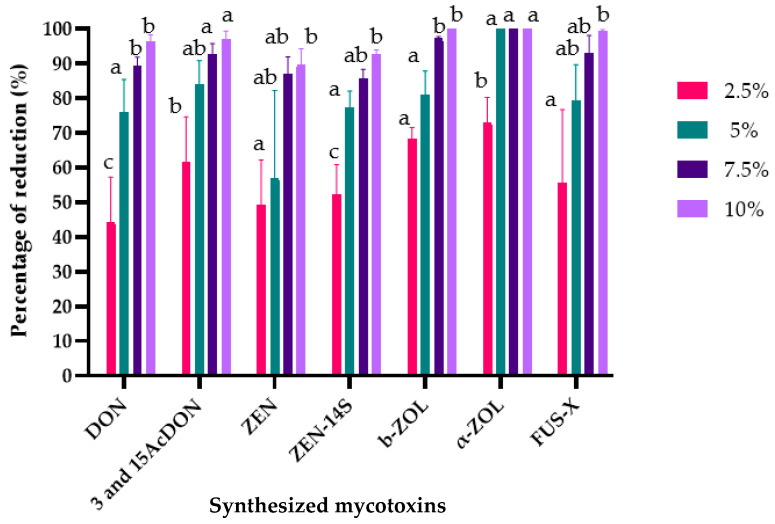
Effects of *L. album* extracts (2.5, 5, 7.5, and 10%) on the production of mycotoxins by *F. culmorum*. The average with different letters (a–c) for each mycotoxin is significantly different at *p* < 0.01. Error bars represent standard deviation (n = 3).

The effects of *L. album* flower extracts on mycotoxin biosynthesis were assessed using a multi-mycotoxin analysis method. The results showed that 12 different mycotoxins out of 22 examined were identified and quantified. In relation to the control samples (PDA without extracts), all tested concentrations of *L. album* flower extracts reduced mycotoxins’ biosynthesis (Figure 3 and Figure 4). The suppressive effect was variable and depended on extract concentration and *Fusarium* isolates. Additionally, the results showed that the 10% concentration was the most effective in reducing mycotoxin biosynthesis in both strains.

*F. proliferatum* synthesized FBs (FB_1_, FB_2_, FB_3_) and beauvercin (BEA), which amounted in the treated samples to the extracts differing significantly at *p* < 0.01, except for FB_2_ (Supplementary Materials Table S1). The increase in extract concentration led to a more significant reduction in mycotoxin concentration (Figure 3). Specifically, BEA was reduced by a maximum of 86.76%, FB_1_ by 87%, FB_2_ by 81%, and FB_3_ by 90%.

Similarly, various mycotoxins, including trichothecenes (DON, 3-AcDON, and 15-AcDON) as well as ZEN and its derivatives (zearalenone-14-sulfate (ZEN-14S); β-zearalenone (β-ZOL); and α-zearalenone (α-ZOL)) and fusarenone-X (FUS-X), were produced by the inoculated *F. culmorum* strain in the treated samples and the control samples but at different concentrations (Supplementary Materials, Table S2). *L. album* flower extracts at the studied concentrations (2.5, 5, 7.5, and 10%) significantly reduced the mycotoxin biosynthesis (Figure 4). The reduction percentages of these mycotoxins were as follows: DON (44.39–96.41%), 3- and 15-AcDON (61.64–96.94%), ZEN (49.38–89.71%), ZEN-14S (52.33–92.61%), β-ZOL (55.57–99.86%), α-ZOL (68.42–100%), and FUS-X (55.57–99.51%).

## 3. Discussion

Management of *Fusarium* pathogens is one of the crucial challenges to minimizing yield loss during harvesting and post-harvest storage of various crops. *Fusarium* species are the common cause of several diseases in wheat, maize, rice, rye, and barley and lead to the accumulation of mycotoxins in grains and other plant tissues, compromising their suitability for human and animal consumption [1,4,6]. Of note is that synthetic fungicides have historically played a crucial role in combating fungi and have benefited crop protection for both small-scale and industrial farmers. However, their use is increasingly restricted or discouraged due to various concerns, including environmental impact, contamination of drinking water, and implications for human health and livestock resulting from improper or excessive use [16]. Therefore, using plant-based fungicides against fungal pathogens can help mitigate the development of resistance, primarily due to the presence of diverse antimicrobial compounds and their synergistic effects. Plant extracts are generally safe, exhibit minimal human toxicity, and are environmentally friendly [12,15]. In contrast to typical synthetic fungicides, their inherent instability at higher temperatures makes them easily biodegradable, ensuring they do not persist in the environment for extended periods [12], and antifungal activities of plant extracts towards *Fusarium* have been reported both in laboratory media and plant matrices [10,30,31,32,33,34,35].

*L. album* is recognized for its diverse bioactive compounds with various biological properties [10,26]. Therefore, it may be considered a natural source of fungicidal compounds.

Results of this study indicate that the *L. album* flower extracts obtained by SC-CO_2_ inhibit mycelium growth and reduce ergosterol and mycotoxins biosynthesis in both *F. culmorum* and *F. proliferatum*.

Though several studies report the antifungal effectiveness of various plant extracts against *Fusarium* spp. [31,32,33,34,35,36,37,38,39,40,41], to the best of our knowledge, there are no existing data concerning the antifungal potential of *L. album* flower extracts against *Fusarium* spp. The observed antifungal and anti-mycotoxigenic effects of *L. album* flower extracts can be attributed to the group of bioactive compounds, including phenylpropanoids, flavonoids, iridoids, and phenolic acids, potentially present in the extracts [21,22,23,24,25,26,27,28]. These effects could arise from synergistic interactions among diverse phytochemicals found within *L. album* flowers, such as ferulic, *p*-coumaric, gallic acid, myricetin, pinostrobin, and caffeic acid, each with distinct biological properties [21,23,26,29,42]. Furthermore, one of the ongoing efforts is focused on identifying and characterizing specific compounds responsible for the observed antifungal effects of the analyzed extracts. Importantly, our prior study has characterized the antioxidant activity and phenolic compounds, including myricetin, chrysin, and trans-3-hydroxycinnamic acid, indicating that the extraction conditions employed here were optimal for obtaining bioactive compounds of high antioxidant activity [10].

Additionally, antifungal attributes of specific bioactive compounds in various plant extracts have been reported [41,42,43,44]. For instance, a study conducted by Abhishek et al. (2015) investigated the antifungal effect of *Solanum torvum* leaves against different fields and storage fungi. By sequentially extracting the leaves, researchers isolated a bioactive compound called torvoside K from the chloroform extract. Torvoside K demonstrated substantial antifungal activity against the tested fungi, as evidenced by inhibitory zones ranging from 33.4 to 87.4%. Furthermore, the compound exhibited a concentration-dependent anti-mycotoxigenic effect, ultimately reducing the presence of FB_1_ in both in vitro and in vivo experiments [41]. The studies have also proven that plant extracts’ antifungal potential depends not only on the main components and their concentration but also on compounds found at lower concentrations and their synergistic action. Chen et al. (2018) evaluated the antifungal activity of *Curcuma longa* extract against *F. graminearum*. Eight compounds were identified, and all had inhibitory effects on *F. graminearum* mycelium growth. Curdione showed the highest inhibition of 52.9%, and combining curdione with the other seven compounds greatly enhanced the inhibition, reaching up to 100% [43].

The inhibitory effects of *L. album* flower extracts were concentration-dependent for *F. culmorum*, while *F. proliferatum* did not exhibit a similar trend. However, the highest antifungal activity for both strains was observed at a 10% concentration. These observations align with previous research findings that highlighted the varying impacts of natural plant extracts on fungal growth (Table 3).

*L. album* extracts obtained by SC-CO_2_ at 10% showed comparable or inferior inhibitory effects to other plant extracts across different concentrations and extraction methods, as indicated in Table 3. Reyes-Vaquero et al. (2021) showed that one plant could inhibit two different species differently (*Ruta graveolens* on *F. oxysporum* and *F. proliferatum*) [31]; similarly, Kursa et al. (2022) observed different inhibitory effects of *Salvia officinalis L.* (sage) against the three studied *Fusarium* strains [34]. This highlights the plant-specific nature of antifungal activity. Furthermore, Ngegba et al. (2018) reported that three different plants at the same concentration inhibited one fungal species differently (*Azadirachta indica*, *Tithonia diversifolia*, and *Chromolaena odorata* on *F. oxysporum*) [32]. This suggests that the effectiveness of plant extracts is influenced by the unique properties of each plant material. Therefore, the diverse antifungal effects summarized in Table 3, along with the results of our study, underscore the complexity of plant–fungal interactions, emphasizing the need for a nuanced understanding of each plant’s bioactivity against specific fungal pathogens.

The assessment of ergosterol content further supported the inhibitory effects of the *L. album* extracts. The results showed a significant reduction in ergosterol content in the treated samples compared to the control group, with reductions ranging from 24.17 to 88.87% for *F. culmorum* and 61.56 to 93.17% for *F. proliferatum*, which may be explained by significant disruption of the fungal cell membrane [43]. Bodoira et al. (2020) have shown that the extracts obtained from agro-industrial by-products of peanut, sesame, and pistachio reduced ergosterol content by 25, 66, and 33%, respectively, compared to the control [35]. However, it should be noted that the reduction in ergosterol content does not always correlate with mycotoxin reduction and vice versa [37,38]. This conflicting outcome arises from the potential unintended consequences of employing biological fungicides; instead of inhibiting fungal growth and mycotoxin biosynthesis, they may boost mycotoxin production [35,39].

A significant reduction in mycotoxins in both tested fungi was observed with increased *L. album* flower extract concentrations. These results align with previous studies on the inhibitory effects of reducing mycotoxin biosynthesis using natural plant extracts [40,41]. It is essential to highlight that these two strains synthesize distinct mycotoxins and do not share any common types, as the multi-mycotoxin analysis method showed. Heidtmann-Bemvenuti et al. (2016) investigated the antifungal activity of natural compounds, including *γ*-oryzanol, a phenolic extract of neem seeds and rice bran, on three toxigenic strains of *Fusarium graminearum* isolated from wheat, rice, and barley. Phenolic extracts of rice bran and neem seeds completely inhibited ZEN production and retarded NIV production. On the other hand, *γ*-oryzanol demonstrated a notable ability of inhibiting ZEN, achieving complete inhibition (100%) in the wheat and rice isolates. Thus, the phenolic extracts were more effective than *γ*-oryzanol in inhibiting the *F. graminearum* strains [40].

Among the mycotoxins produced by *F. proliferatum*, BEA exhibited the highest prominence. However, its reduction was similar to the reduction observed for other mycotoxins in this study. Similar effects have been reported for natural phenolic compounds such as ferulic acid, sinapic acid, and bromelain, which completely prevented the synthesis of BEA in *F. proliferatum* and reduced it in *F. ananatum* strains [45]. Current studies indicate a high prevalence of BEA in grains and wheat-based products such as pasta, infant formulas, breakfast cereals, and biscuits, with incidence rates ranging from 40 to 90% [46]. However, major food regulatory bodies like the US Food and Drug Administration and the European Food Safety Authority have not yet established specific regulations or guidelines for permissible levels of emerging mycotoxins, including BEA [47].

The *F. culmorum* strain produced different mycotoxins. Among them, ZEN-14S was the most prominent mycotoxin produced by this strain. ZEN-14S is a modified form of ZEN synthesized by different *Fusarium* species [48]. The results revealed that its reduction aligned with that of the other mycotoxins. Previous research has suggested the possibility of its transformation into free forms [49], a phenomenon not observed in our study. Therefore, the identification and characterization of a diverse set of mycotoxins underscore the importance of analyzing all possible mycotoxins produced by tested strains to avoid an incorrect assessment of the mycotoxin content, which poses potential health risks to consumers [13]. This study demonstrated that *L. album* flower extracts can inhibit not only well-known mycotoxins but also their modified forms and derivatives.

## 4. Conclusions

Applying plant extracts as a source of natural compounds against phytopathogenic and mycotoxigenic fungi presents an interesting approach, primarily as the world seeks sustainable solutions for environmental problems and food safety. The conducted experiments revealed promising in vitro effects of *L. album* flower extracts against *F. culmorum* and *F. proliferatum,* which are significant pathogens affecting cereals. The extracts inhibited the growth of these fungi significantly better at a higher concentration (10%) compared to lower concentrations (2.5, 5, and 7.5%). These inhibitory effects were further confirmed by reduced amounts of ergosterol and mycotoxins. The overall effects of *L. album* flower extracts obtained by SC-CO_2_ depended on the extract’s concentration, type of strain, and the biosynthesized mycotoxins. These findings offer valuable insights for future research endeavors to characterize the primary active components in the extracts derived from *L. album* flowers. Additionally, a comprehensive in vivo study will be essential to practically evaluate the effectiveness of *L. album* flower extracts against *Fusarium* pathogens in cereal crops and to gain insight into their inhibitory mechanisms. This ongoing effort holds significant promise for promoting sustainable agricultural practices, addressing the challenges related to fungal diseases in cereal crops, and contributing to the positive enhancement of food safety.

## 5. Materials and Methods

### 5.1. Chemicals

Carbon dioxide (CO_2_, SFE grade), contained in a dip tube cylinder, was purchased from Air Products Sp, Poland. Methanol for HPLC-super gradient was purchased from POCh (Gliwice, Poland). Acetonitrile, methanol, and water for LC-MS grade were acquired from POCh (Gliwice, Poland). Oxoid (Basingstoke, UK) supplied PDA. All chemicals were of analytical grade. Analytical standards purchased in ready-to-use solutions from Romer Labs (Tulln, Austria) included: ERG, FB_1_, FB_2_, FB_3_, ZEN, DON, 15-AcDON, 3-AcDON, and BEA, which were 100 µg/mL. The β-ZOL concentration was 10 µg/mL. ZEN-14S (100 µg/mL) was purchased in Aokin (Berlin, Germany). Depending on solubility, the standards were dissolved in acetonitrile. All standards were stored in amber glass vials at approximately minus 20 °C. A mixture of all standards necessary for a particular analytical run was prepared immediately before the analysis. 

### 5.2. Plant Material

High-quality dried *L. album* flowers *(Lamii Albi flos)* were purchased from a certified Polish company called Dary Natury located in Podlaskie Voivodeship of Poland (53°4′10.98 latitude and 22°58′2.87 longitude).

### 5.3. Lamium album Flower Extraction

The extracts were obtained using a supercritical CO_2_ extraction method with methanol as a co-solvent (MV-10 ASFE, Waters, Manchester, MA, USA) [26]. The fine-ground samples of *L. album* flowers (9 g) were loaded in the extraction vessel of 25 mL and inserted in the oven set at the desired temperature (50 °C) and pressure (250 bar). The CO_2_ flow rate was 4 mL/min, and the methanol flow rate was fixed at 1 mL/min. The extraction time was 180 min, divided into 1st dynamic time of 45 min, a static time of 15 min, and 2nd dynamic time of 120 min. *L. album* extracts were collected in flasks placed in a fraction collection module. The obtained methanolic extracts were transferred into a round bottom flask and evaporated in a vacuum evaporator (Buchi R-215 Rotary Evaporator System, Essen, Germany) to eliminate the methanol; the dried extract was reconstituted with distilled water and stored at −18 °C until it was required for the antifungal assay.

### 5.4. Description of Fungal Test Pathogens

Isolates of two plant pathogenic *Fusarium* species, *F. proliferatum* PEA 1 and *F. culmorum* KF 846, were used as test pathogens. The strains were initially isolated from pea seeds and wheat kernels, respectively. They were identified by molecular techniques [50], stored in the collection of pathogenic fungi at the Institute of Plant Genetics, Polish Academy of Sciences, Poznan, Poland, and had already been characterized during previous research.

### 5.5. Determination of the Antifungal Activity of L. album Flower Extracts

The study evaluated the antifungal effects of 2.5, 5, 7.5, and 10% of *L. album* flower extract against *F. culmorum* and *F. proliferatum* growth. For this objective, the poisoned food technique was used [41,51]. This technique involves mixing the prepared *L. album* extract with a cooled PDA (45 °C) medium. Fifteen ml of PDA medium/extract-supplemented medium was poured onto each plate using an automatic dispenser. Using a sterile cork borer, agar discs with mycelia (6 mm in diameter) were cut from the periphery of actively growing regions of the 7-day-old pure cultures and aseptically inoculated at the center of the Petri plates. The controls contained only PDA medium and fungal discs in the middle. All the inoculated plates were incubated at 25 °C in the dark for 10 days, and the radial mycelial growth was measured after 3, 6, and 10 days. Triplicates were maintained for each concentration and control. The experiment was repeated three times. Finally, the antifungal activity of each extract concentration was calculated in terms of the inhibition percentage of mycelia growth using the following formula reported in previous studies [10,41].

The inhibition percentage of fungal growth (%) = [(C − L)/C] × 100(1)

where C is the average diameter of fungal growth on control Petri dishes, and L is the average diameter of fungal growth on Petri dishes with *L. album* flower extracts. Following the incubation period, the contents of each petri dish (PDA with mycelia) were collected and lyophilized. Subsequently, the dried samples were ground into a fine powder and stored at room temperature for subsequent experiments, including the quantification of ERG and mycotoxins.

### 5.6. Determination of ERG Content

For the ERG quantitation, the procedure described by Uwineza et al. [10] was followed. The dried PDA with mycelium (100 mg) was suspended in 2 mL of methanol in a culture tube and treated with 0.5 mL of 2 M aqueous sodium hydroxide. Samples were irradiated three times in a microwave oven (370 W) for 10 s and then were neutralized with 1 mL of 1 M aqueous hydrochloric acid. Samples were extracted with n-pentane (3 × 4 mL) and transferred to the vials. Extracts were evaporated to dryness in a stream of nitrogen. Before chromatographic analysis, dry residues were dissolved in 1 mL of methanol. The ERG separation was performed on a 3.9 mm Nova Pak C-18, 4 mm column with methanol:acetonitrile (90:10, *v*/*v*) as the mobile phase at a 1.0 mL/min flow rate. ERG was detected with a Waters 2996 Photodiode Array Detector (Waters Division of Millipore, Milford, MA, USA) set at 282 nm. The presence of ERG was confirmed by comparing retention times with the external standard and by co-injection of every tenth sample with an ERG standard. The detection limit was 10 ng/g.

### 5.7. Determination of Mycotoxins by Using UPLC/MS/MS

The mycotoxins were extracted by adding 5 mL of the extraction solvents (acetonitrile:water, 86:16, *v*/*v*) to 0.5 g of dried PDA with mycelium; the extraction process involved vortexing for approximately 30 s and mixing with a horizontal shaker for 24 h. Then, the samples were centrifuged at 10,000 rpm for 10 min. After centrifugation, approximately 2 mL of mycotoxin extracts were filtered through a 0.45 µm nylon syringe filter and collected in vials for mycotoxin identification and quantification. For the analysis, the method reported by Uwineza et al. (2022) was followed with some modifications [10]. The analytes were quantitatively analyzed using multiple reaction monitoring, and their identification was confirmed by comparing the retention times and *m*/*z* values with those of corresponding standards. The analysis was performed in triplicate. 

### 5.8. Statistical Analysis 

The results are presented as the mean (±) standard deviation of three parallel replicates. A one-way analysis of variance was performed to evaluate the significance of differences in the *L. album* flower extracts (2.5, 5, 7.5, and 10%) in the determined ERG and mycotoxin concentrations in PDA mycelium. Subsequently, a post hoc Tukey’s honest significant difference (HSD) test with a significance level of α = 0.01 was conducted for paired comparisons. Analyses were conducted using the Statgraphics v. 4.1 software package (Graphics Software System, STCC, Inc., Rockville, MD, USA) and GraphPad Prism 9 software.

## Figures and Tables

**Table 1:** The inhibitory effect of *L. album* extracts (2.5, 5, 7.5, and 10% concentration) on the mycelium growth of *F. culmorum* and *F. proliferatum*.

Extract Concentration (%)	Mycelium Growth Inhibition (%)	
	*F. culmorum*	*F. proliferatum*
Control without extracts	-	-
2.5	0.00 ^a^	27.71 ± 6.26 ^b^
5	0.00 ^a^	40.16 ± 0.70 ^a^
7.5	23.53 ± 2.04 ^b^	40.56 ± 0.70 ^a^
10	30.59 ± 2.04 ^c^	42.97 ± 0.70 ^a^

All values are means of three replicates ± standard deviation. The superscripts of different letters in rows are significantly different (Tukey’s HSD test, significant at *p* < 0.01).

**Table 2:** Effects of *L. album* extracts (2.5, 5, 7.5, and 10%) on ergosterol (ERG) content (µg/g) and its reduction (%) after 10 days of incubation at 25 °C on a PDA medium inoculated with *Fusarium* species.

Extract Concentration (%)	ERG Concentration (µg/g) and Percentage of Reduction (%)			
	*F. culmorum*		*F. proliferatum*	
	(µg/g)	(%)	(µg/g)	(%)
Control (without extracts)	5036.19 ± 1178.93 ^c^	-	20,234.01 ± 1484.40 ^d^	-
2.5	3818.97 ± 829.56 ^bc^	24.17 ^c^	7778.33 ± 227.47 ^c^	61.56 ^c^
5	1864.53 ± 706.63 ^ab^	62.98 ^a^	4278.58 ± 281.07 ^b^	78.85 ^b^
7.5	1016.00 ± 200.36 ^a^	79.83 ^ab^	1549.54 ± 157.36 ^a^	92.34 ^a^
10	560.59 ± 140.09 ^a^	88.87 ^b^	1381.55 ± 191.88 ^a^	93.17 ^a^

All values are means of three replicates ± standard deviation. The superscripts of different letters in rows are significantly different (Tukey’s HSD test, significant at *p* < 0.01).

**Table 3:** The comparative assessment of the inhibitory effect of different plant extracts against *Fusarium* spp.

Plant Material	Extraction Method	Conc *	Antifungal Assay	*Fusarium* spp.	Inhibitory (%)	Ref. *
*Ruta graveolens*	Solid–liquid method ethanol:water (85:15 *v*/*v*)	16 mg/mL	Agar dilution method	*F. oxysporum*	72.90	[31]
				*F. proliferatum*	68.73	
*Azadirachta indica*	Distilled water	100 mg/mL	-	*F. oxysporum*	87.40	[32]
*Tithonia diversifolia*					76.80	
*Chromolaena odorata*					62.70	
*Eucalyptus camaldulensis* leaves	Distilled water	4 mg/mL	Agar dilution method	*F. solani*	78.58	[33]
				*F. oxysporum*	77.80	
*Salvia officinalis* leaves	Reflux condenser (70% ethanol)	20%	Poisoned method	*F. avenaceum*	61.30	[34]
				*F. culmorum*	52.59	
				*F. graminearum*	38.39	
*Solanum torvum* leaves	Solvent extraction (chloroform)	1 mg/mL	Poisoned food technique	*F. verticillioides*	76.42	[41]
				*F. oxysporum*	68.00	

Conc * = concentration, Ref * = reference.

## Data Availability

Data are contained within the article and supplementary materials.

## Supplementary Materials

The following supporting information can be downloaded at: https://www.mdpi.com/article/10.3390/toxins15110651/s1, Table S1. The effect of different concentrations of *L. album* flower extracts (2.5, 5, 7.5, and 10%) on *F. proliferatum* mycotoxins after 10 days of incubation at 25 °C on a PDA medium; Table S2. The effect of different concentrations of *L. album* flower extracts (2.5, 5, 7.5, and 10%) on *F. culmorum* mycotoxins after 10 days of incubation at 25 °C on a PDA medium.
