# Supplementary material for: Lamium album Flower Extracts: A Novel Approach for Controlling Fusarium Growth and Mycotoxin Biosynthesis
Source: Toxins (Basel). 2023 Nov 13;15(11):651. doi: 10.3390/toxins15110651 (PMC10675686; doi:10.3390/toxins15110651)
Supplement: Supplementary file 1 [file toxins-15-00651-s001.zip › toxins-2634042-supplementary.pdf]

toxins

MDPI

Table S1. The effect of different concentrations of *L. album* flower extracts (2.5, 5, 7.5, and 10%) on *F. proliferatum* mycotoxins after 10 days of incubation at 25 °C on a PDA medium.

**Extract concentrations [%]**	**Mycotoxins level [μg/g]**			
	**FB₁**	**FB₂**	**FB₃**	**BEA***
Control (without extracts)	59.35 ± 15.22c	4.63 ± 1.42ab	0.83 ± 0.07b	118.78 ± 19.04c
2.5	48.28 ± 9.07bc	4.44 ± 0.59ab	0.42 ± 0.17ab	77.84 ± 21.35bc
5	39.40 ± 14.32abc	3.19 ± 1.09 ab	0.30 ± 0.26ab	51.77 ± 14.18ab
7.5	15.59 ± 7.13ab	2.26 ± 1.34ab	0.21 ± 0.12a	36.10 ± 4.87ab
10	7.24 ± 1.75a	0.81 ± 0.19 a	0.08 ± 0.04a	17.98 ± 7.82a

All values are means of three replicates ± standard deviation. The superscripts of different letters in rows are significantly different (Tukey's HSD test, significant at $p < 0.01$).

Table S2. The effect of different concentrations of *L. album* flower extracts (2.5, 5, 7.5, and 10%) on *F. culmorum* mycotoxins after 10 days of incubation at 25 °C on a PDA medium.

**Extract Concentrations [%]**	**Mycotoxins* level [µg/g]**						
	**DON**	**3- + 15-AcDON**	**ZEN**	**ZEN-14S**	**β-ZOL**	**α-ZOL**	**FUS-X**
Control (without extracts)	165.35 ± 51.76 ^{b}	201.69 ± 43.77 ^{b}	592.95 ± 106.71 ^{b}	887.08 ± 95.32 ^{c}	1.94 ± 0.67 ^{b}	0.28 ± 0.10 ^{b}	6.23 ± 1.03 ^{b}
2.5	87.54 ± 8.75 ^{ab}	73.77 ± 8.38 ^{a}	293.49 ± 52.23 ^{a}	425.09 ± 100.21 ^{b}	0.62 ± 0.24 ^{a}	0.07 ± 0.01 ^{a}	2.74 ± 1.37 ^{a}
5	36.683 ± 5.77 ^{a}	30.80 ± 12.35 ^{a}	237.22 ± 91.18 ^{a}	199.01 ± 31.91 ^{ab}	0.35 ± 0.14 ^{a}	nd* ^{a}	1.27 ± 0.64 ^{a}
7.5	17.43 ± 6.33 ^{a}	14.30 ± 4.18 ^{a}	73.64 ± 20.33 ^{a}	128.87 ± 36.09 ^{a}	0.05 ± 0.01 ^{a}	nd ^{a}	0.45 ± 0.37 ^{a}
10	5.3 ± 2.05 ^{a}	5.45 ± 3.35 ^{a}	58.06 ± 19.37 ^{a}	64.76 ± 6.56 ^{a}	nd ^{a}	nd ^{a}	0.03± 0.02 ^{a}

All values are means of three replicates ± standard deviation. The superscripts of different letters in rows are significantly different (Tukey's HSD test, significant at $p < 0.01$), *nd—not detected.
